# Simulation of Swanson's Literature-Based Discovery: Anandamide Treatment Inhibits Growth of Gastric Cancer Cells *In Vitro* and *In Silico*

**DOI:** 10.1371/journal.pone.0100436

**Published:** 2014-06-20

**Authors:** Weiwei Dong, Yixuan Liu, Weijie Zhu, Quan Mou, Jinliang Wang, Yi Hu

**Affiliations:** 1 Department of Oncology, General Hospital of PLA, Beijing, China; 2 Department of Gastroenterology, Peking University Third Hospital, Peking University Health Science Center, Beijing, China; 3 Beijing Centers for Diseases Control and Prevention (CDC) and Centers for Preventive Medical Research, Beijing, China; 4 Department of Surgery, PLA 252 Hospital, Baoding, China; Centro di Riferimento Oncologico, IRCCS National Cancer Institute, Italy

## Abstract

Swanson's literature-based discovery focus on resurrecting previously published but neglected knowledge. In this study, we propose a two-step model of the discovery process and generate a hypothesis between anandamide and gastric cancer. Further, the potential relationship was confirmed by follow-up experimentation. The anandamide treatment resulted in cell cycle redistribution of gastric cancer cells. Most importantly, the variation of cell cycle was mediated by some genes from the B-terms of the closed discovery, indicating the potential role of the B-terms. Swanson's literature-based discovery not only collates data for possible interactions, but also provides the potential to observe the larger background behind these direct links and is an invaluable discovery tool for investigators.

## Introduction

The rapid development of science has led to an exponential growth in our knowledge base. Consequently, it is very challenging for medical investigators to retrieve and incorporate the most relevant, updated information from the daily deluge of published literature. For example, the total number of publications indexed by the search term ‘gastric cancer’ increased more than 4-fold in 2012 compared to 1980. Often, the most relevant information tends to be overlooked or neglected in current studies [1]. Additionally, with increasing specialization in the areas of science and technology, greater challenges exist in sharing information cross-functionally, as scientific understanding is becoming more focused and less diversified. However, a meaningful relationship must exist among the different research fields. Therefore, it is more important to determine the ignored knowledge than the information growth itself [2].

Swanson (1990) developed and implemented a novel tool to mine the existing knowledge base for unreported or underreported relationships, and resurrect previously published but neglected hypotheses, a process known as literature-based discovery [3]. This process functions as a way of connecting 2 seemingly unrelated findings, otherwise stated in this form: if “A is related to B” and “B is related to C”, then the hypothesis that “A causes C” is strongly suggested [4]. Although this approach does not provide a conclusive proof, the discovery is, in itself, very helpful in uncovering previously unknown relationships [5]. Further, it can help the investigators access context and mine knowledge that might not be revealed using a traditional search.

In the present study, we performed a 2-step approach to simulate Swanson's literature-based discovery methodology in 2 fields of biological research literature that are normally not bibliographically connected: gastric cancer and psychology. Gastric cancer is one of the most commonly diagnosed cancers, the second leading cause of cancer-related death worldwide, and a serious public health problem [6]. Previous research has also demonstrated that a variety of psychological factors can have a considerable effect on physical diseases. However, the relationship between gastric cancer and psychology has previously been neglected. Therefore, these 2 fields of research were selected with the goal of finding a neglected common connection.

The 2-step discovery process generated a hypothesis about the correlation between anandamide and gastric cancer and provided a possible common molecular network which may mediate the effects. The hypothesis was then investigated experimentally. Anandamide was found to inhibit growth in 4 gastric cancer cell lines, including BGC823, SGC7901, AGS, and N87. Flow cytometry data demonstrated that the presence of anandamide induced G2/M cell cycle arrest in AGS and N87 cells. Furthermore, we confirmed that anandamide can act to regulate cell cycle associated genes, including CHEK1, CDKN1A, CDKN2A, obtained from the closed discovery process. Collectively, these data indicate that anandamide affects the cell cycle distribution of gastric cancer cells by regulating the B-terms cell cycle regulators, a hypothesis that was validated experimentally in this study, thereby providing investigators with an alternate view regarding the role of anandamide. Using this approach, we were able to successfully piece together a previously hidden relationship between 2 disparate fields and add to the overall knowledge base.

## Methods and Materials

### The articles in MEDLINE database

The National Institutes of Health (NIH)-sponsored MEDLINE/PubMed database contains links to articles published in over 5500 leading journals. To acquire the most complete series of articles for “gastric cancer,” an inclusive series of search strings (Neoplasm; Stomach or Stomach Neoplasm or Neoplasms; Stomach or Neoplasms; Gastric or Gastric Neoplasms or Gastric Neoplasm or Neoplasm; Gastric or Cancer of Stomach or Stomach Cancers or Stomach Cancer or Cancer; Stomach or Cancers; Stomach or Cancer of the Stomach or Gastric Cancer or Cancer; Gastric or Cancers; and Gastric or Gastric Cancers or Stomach Neoplasms) was used to retrieve the appropriate articles. This search retrieved 86,536 records (accessed on October 27, 2011), which included all publications entered into this database. Additionally, to acquire the most complete series of articles for “psychology,” an inclusive series of search strings (Psychology or Side Effects; Psychological or Psychological Side Effects or Psychological Side Effect or Side Effect; Psychological or Psychosocial Factors or Factor; Psychosocial or Factors; Psychosocial or Psychosocial Factor or Psychological Factors or Factors; Psychological or Factor; and Psychological or Psychological Factor or Psychologists or Psychologist) retrieved 911,793 records. All of these publications were saved in Medline format for collecting Medical Subject Headings (MeSH) terms.

### MeSH terms

MeSH is the controlled vocabulary thesaurus of the National Library of Medicine. MeSH consists of sets of terms called descriptors that are arranged in both alphabetical and hierarchical structures, therefore, MeSH terms can identify the primary focus of the publication, and may be used to index Medline records. In this study, these descriptors were filtered using a MeSH stoplist of 5,569 descriptors specific to the subject areas of this study.

### Stoplists

A stoplist is a list of words that should be excluded, either because they are too broad or are meaningless as targets for a search. Stoplists may include as many as several hundred functional words that are not subject oriented. In this study, a long stoplist of 5,569 words was compiled to filter the MeSH terms. Using MeSH categories provided by the Medline database and investigator judgment, we compiled lists of stop-terms, with the top-level MeSH categories displayed in Table 1. Detailed information on MeSH categories can be found at http://www.nlm.nih.gov/mesh/2012/mesh_bro-wser/MeSHtree.Dhtml.

**Table 1:** The top-level MeSH categories of the stop-terms list.

Filtratable Top-level MeSH Categories
Anatomy [A] (A01-A11)
Analytical, Diagnostic, and Therapeutic Techniques and Equipment[E] (E04-07)s
Anthropology, Education, Sociology, and Social Phenomena [I] (I1-I2)
Technology, Industry, and Agriculture [J]
Humanities [K]
Information Science [L]
Named Groups [M]
Publication Characteristics [V]
Geographicals [Z]

### Open discovery process

The open discovery process begins with a scientific problem and the generation of a hypothesis. In this study, the fields of gastric cancer and psychology were selected to search for hidden relationships. We retrieved the publications relating to gastric cancer and psychology as described previously. Next, the MeSH profile was constructed for initiating the A or B topic. We constructed topic profiles by identifying a relevant subset of characteristic terms from the selection of text. Terms were extracted from the documents and assessed for their relative importance as descriptors of the topic. In this study, our profiles were weighted vectors of MeSH terms, as shown below for a topic X_i_:



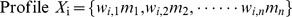
(1)where m_j_ represents a MeSH term, w_i,j_ represents its weight, with a total of n terms in the MeSH vocabulary [7].

After topics A and B were constructed, the final combination—a C profile—was constructed using a Java applet that we developed. In this procedure, terms appearing in both A and B profiles were eliminated, together with terms that were only present in the A profile. Finally, the MeSH terms that were only present in the B profile and rarely appeared in the A profile were selected and called the C profile. The terms in the C profile showed close connections with topic B and few or no links to topic A. In other words, these concepts remained closely associated with “psychology,” but were rarely reported together with “gastric cancer.” However, previously unreported connections may exist, which may be uncovered using the subsequent procedure.

### Closed discovery process

The closed discovery process was performed using the web-based model Arrowsmith (http://arrowsmith.psych.uic.edu/arrowsmith_uic/index.html; University of Illinois, Chicago, Department of Psychiatry), which is designed to assist with comprehension and recognition of hidden connections between the A and C literature components [8], [9]. We initiated the closed discovery process with the first 2 Medline searches using the Arrowsmith processing: A topic profile search and C term literature search. Next, the Arrowsmith program generated a list of terms that could be found in the titles of both A and C literature retrievals. These words were analyzed to generate a B-list, using a third filtering step, by:

- selecting certain semantic categories (Activities & Behaviors; Anatomy; Genes & Molecular Sequences, and Gene & Protein Names; Chemicals & Drugs; Physiology);
- removing B-terms that appeared fewer than 5 times in either literature;
- keeping only B-terms that first appeared in the last 10 years;
- removing B-terms having literature cohesion scores less than 0.1.

Finally, we generated our list of B-concepts and then viewed the corresponding AB and BC literature retrievals.

### Cell culture

The cell lines used in present study were all gastric cancer cell lines. The N87 and AGS cell lines were obtained from the American Type Culture Collection (ATCC, Manassas, VA, USA). AGS cell line was derived from fragments of a tumor resected from a patient who was a 54-year-old Caucasian female and had received no prior therapy. N87 cell line was derived from a liver metastasis of a well differentiated stomach carcinoma of a Caucasian male taken prior to cytotoxic therapy. AGS and N87 cells were cultured in Dulbecco's modified Eagle's medium (DMEM; Invitrogen, Carlsbad, CA, USA), supplemented with 10% fetal bovine serum (FBS). The cell lines BGC823 and SGC7901 were established in China. The BGC823 cell line was established in 1984 through 60 passages of cell culture in the period of 2 years from a surgical specimen of a stomach cancer patient which was diagnosed as low differentiated adenocarcinoma. SGC7901 cell line was established in 1979 and derived from a lymphatic metastasis of gastric adenocarcinoma of a 56-year-old Chinese female. The BGC823and SGC7901 cells were cultured in Dulbecco's modified Eagle's medium (DMEM; Invitrogen, Carlsbad, CA, USA), supplemented with 5% fetal bovine serum (FBS); All cell lines were maintained at 37°C in 5% CO_2_.

### Flow cytometric analysis of cell cycle status and apoptosis

The AGS and N87 cells (1×10^5^) were routinely harvested by trypsin digestion after treatment with anandamide (Sigma, A0580), washed with PBS, and fixed in cold 75% ethanol at 4°C overnight. After staining with propidium iodide (PI) solution for 30 min, cell cycle analysis was performed by fluorescence activated cell sorting (FACS) to determine the percentage and distribution of cells in the G1, S, and G2/M phases.

For detection of phosphatidylserine externalization, trypsinized cells were double-stained with fluorescein isothiocyanate (FITC)-conjugated Annexin V (20 µg/mL) and PI (50 µg/mL) according to the manufacturer's protocol (Biosea; Nelson, New Zealand). Ten thousand cells were collected and analyzed by FACSorter, and the cell cycle profiles were analyzed using Cell Quest software (Becton-Dickinson, Franklin Lakes, NJ, USA).

### MTT assay

BGC823, SGC7901, AGS, and N87 cells were plated into 96-well culture plates and incubated with anandamide at different concentrations, followed by 3-(4,5-dimethylthiazol-2-yl)-2,5-d-iphenyltetrazolium bromide (MTT) at 24, 48, 72, and 96 h. After 4-h incubation at 37°C in 5% CO_2_, 200 µL dimethyl sulfoxide (DMSO) was added to solubilize the formazan product. The absorbance at 570 nm was determined using a microplate reader (Bio-Rad Laboratories; Hercules, CA, USA).

### Gene expression profiling analysis

The 2 different RT2 Profiler PCR Arrays (SA Biosciences, QIAGEN) were used to analyze the expression levels of 84 genes involved in regulation of cell cycle (Human Cell Cycle array, PAHS-020) in AGS cells treated with anandamide or vehicle. The cells were harvested at 24h after anandamide treatment and total RNA was extraction using RNeasy Plus kit (QIAGEN). The synthetic cDNA was used as template to perform quantitative polymerase chain reaction (PCR) analysis. The RT2 profiler PCR Array Data analysis software (v3.5) was used for PCR array data analysis. We selected the GAPDH and ACTB as reference genes. Genes with expression fold changes between samples larger than 2 are selected as differential genes.

### Western blot analysis

Protein lysates were separated by 12% Tris-glycine polyacrylamide gel electrophoresis and transferred to polyvinylidene fluoride (PVDF) membranes (GE Healthcare; Little Chalfont, UK). Membranes were incubated with mouse monoclonal antibody against human Chk1, p-Chk1 (Abcam; Cambridge, UK), CDC25A, CyclinB1 (Santa Cruz Biotechnology, Santa Cruz, CA, USA), followed by horseradish peroxidase (HRP)-labeled goat-anti-mouse IgG (PTGLab; Chicago, IL, USA). β-actin (Sigma Chemicals; St. Louis, MO, USA) was used as a protein loading control. The HRP conjugate was detected by a chemioluminescence ECL kit (Amersham) and autofluorography. Protein intensity was quantified using the LANE 1D Analyzer software (Sage Creation, Beijing, China).

### Statistical analysis

The Student's t-test was used to analyze the significance of difference between two groups of data. P*<*0.05 was regarded as statistically significant.

## Results

### Acquisition of MeSH terms for gastric cancer and psychology

In the open discovery process, the first step was to extract the MeSH terms from the literature retrievals. We generated 12,001 MeSH terms (Profile A) using the text for “gastric cancer.” These MeSH terms involved aspects of gastric cancer that include diagnosis, treatment, medication, survival, cancer stage, cell function, genes, proteins, and some chemical substances like esters. We generated 9,635 MeSH terms (Profile B) using the text for “psychology”, which are closely related to social environment, behavior, personality, feelings, kinds of diseases, therapy, drugs, chemical substance, etc. The top ten MeSH terms from each topic are displayed in Table 2.

**Table 2:** The top 10 MeSH terms of 2 topics—gastric cancer (A) and psychology (B).

Rank	Mesh Terms of Profile A	Frequency	Mesh Terms of Profile B	Frequency
1	Stomach Neoplasms	67564	Psychology	135618
2	Adenocarcinoma	14128	Anxiety	86828
3	Gastrectomy	10296	Affect	86472
4	Gastric Mucosa	6814	Fear	45843
5	Stomach	6577	Depression	36178
6	Neoplasm Staging	6018	Adaptation,Psychological	35363
7	Helicobacter Infections	5308	Stress,Psychological	21565
8	Lymphatic Metastasis	5215	Interpersonal Relations	19861
9	Diagnosis,Differential	4879	Cognition	17416
10	RetrospectiveStudies	4792	Grief	14358

### Discovery of anandamide in the C profile (B-A)

Using the aforementioned Java applet, we eliminated terms appearing in the A profile from the B profile, leaving only terms present in the B profile, but never appearing in the A profile. This new set was identified as the “C profile.” Terms in the C profile were reported to have more than 1 relationship with psychology, but few or no publications simultaneously referencing both psychology and gastric cancer. However, previously unreported connections may exist that have not been discovered, or were ignored.

To further explore the potential relationships between these fields, we classified the terms of the C profile into 6 categories, including behavior, organ and tissue, moods, diseases, drugs and chemical substances, and other. Each term possessed its own frequency; the terms possessing the higher frequencies and representativeness are displayed in Table 3. Finally, we selected 1 term from the drug and chemical substance category, anandamide, for further study. The reasons for our selection are summarized below:

**Table 3:** The 6 categories of the target profile C.

Concepts	Frequency	Concepts	Frequency
**Behavior**		**Drugs or Chemical Substance**	
Hostility	3982	Imipramine	1742
Communication	3936	Diazepam	1382
Arousal	8889	Norepinephrine	907
Alcoholism	3345	Serotonin	801
Marriage	1913	Anandamide	732
**Organ and tissue**		**Diseases**	
Amygdala	3235	Schizophrenia	3257
Hippocampus	2068	Dementia	866
Brain	5924	Agoraphobia	862
Central Nervous system	382	AlzheimerDisease	819
Renal	330	Asthma	815
**Moods**		**Others**	
Anxiety	45843	Frustration	1841
Fear	19861	Loneliness	1898
Depression	17416	Cognition	7219
Anger	5287	Personality	5048
Pain	4797	Family	5046

1. The terms in the drug and chemical category were the easiest to use for experimental testing of the potential link between psychology and gastric cancer.
2. Within the category of drug and chemical, the higher frequency of anandamide indicated its greater relative importance.
3. Other terms with higher frequency, such as diazepam, are all well known, while anandamide is a relatively new substance.

Although few studies were found examining both anandamide and gastric cancer together [10], [11], our work of resurrection focuses on analyzing pathways or mechanisms that might not be known.

### Prediction of relationship between anandamide and gastric cancer

**Figure 1:**
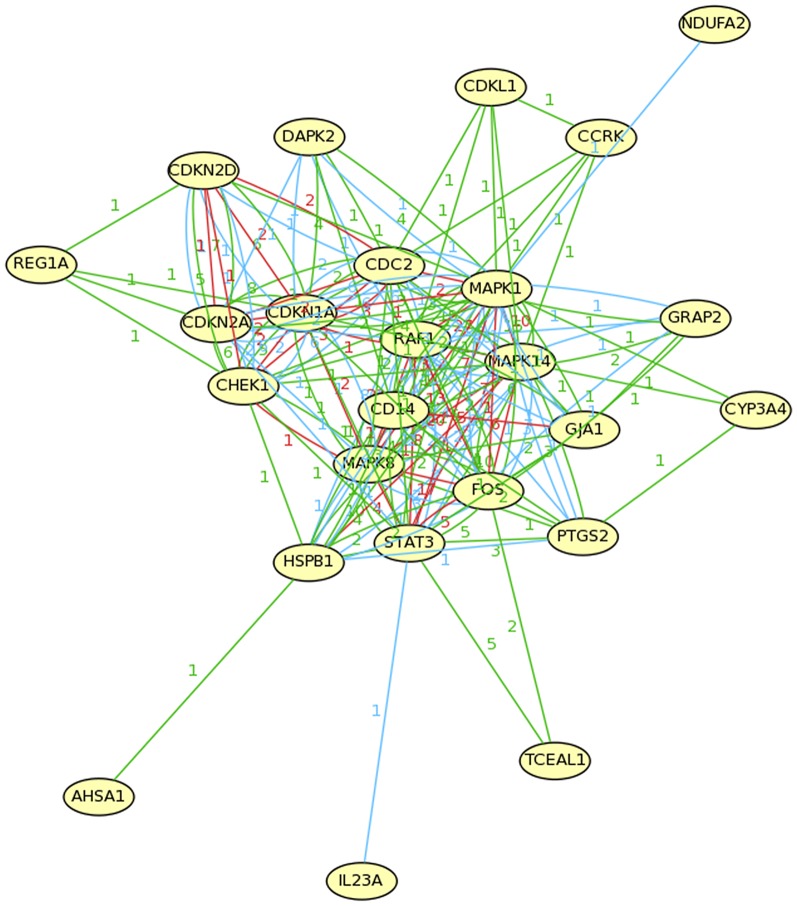
The filtered B-terms made up a molecular regulatory network. The critical genes of the B-terms were collected and using Mas 3.0 annotation system (Capital Bio http://bioinfo.capitalbio.com/mas3/) a molecular network was constructed. The abbreviations represented different genes involved in some important signal pathways related to both gastric cancer and anandamide. e.g., The PTGS2 gene codes for COX-2, an enzyme responsible for inflammation and pain, was reported that its mutationsmay be responsible for a higher risk of gastric cancer, in the meantime, COX-2 also metabolizes anandamide forming prostaglandin-ethanolamides (PG-EA). The numbers and color on the lines connected genes means the counts of the gene appeared in different signal pathways in three biological pathway database including Biocarta, GenMAPP andKEGG (Red: Biocarta; Green:GenMAPP; Blue: KEGG).

Our hypothesis concerning anandamide and gastric cancer was tested through the closed discovery process. This process was performed using the web-based model Arrowsmith; starting from the disease of gastric cancer (A) and the substance of anandamide (C), we searched for common intermediate B-terms. The B-list contained title words and phrases (terms) that appeared in both the A and C literature. As the pathways found between A and C became more common, it increased the likely validity of our hypothesis. Following the necessary aforementioned filter steps, 446 terms were present on the current B-list, ranked according to predicted relevance. Next, we restricted these terms by semantic categories of Genes & Molecular Sequences and Gene & Protein Names and collected the target genes involved in different signaling pathways including cell cycle, apoptosis, inflammation and etc (Table 4). Simultaneously a pathway network was constructed to display the common molecular regulatory network between gastric cancer and anandamide action. As seen in Figure 1, these genes exhibited high dependency indicating the regulatory relationships between them. To further examine the validity of these target genes, several proteins/genes were chosen as examples to verify their relationship to gastric cancer or anandamide through ARROWSMITH SYSTEM. And as shown in Table 5, a group of critical genes involved in several pathways that are linked both to gastric cancer and to anandamide. All of these clues implied a potentially hidden connection between anandamide and gastric cancer, which was worthy of further investigation.

**Table 4:** The B-term genes make up potential common molecular pathways between gastric cancer and anandamide.

Pathway name	Gene symbol		
Cell cycle	CCNA2	CCNB1	CDC25A
	CDC25C	CHEK1	CHEK2
	CCRK	CDKN1A	CDKN2A
	CDC2	CDKL1	CDKN2D
Apoptosis	DAPK2	HSPB1	IGF1R
	AKT	NOD1	STAT3
	CASP2	RIPK2	TCEAL1
RAS/MAPK	MAPK1	MAPK14	MAPK8
	RAF1	FOS	
P53	GADD45A	ATM	ATR
	CDKN1A	CDKN2A	
Inflammation	IL23A	PTGS2	AHSA1
	STAT3	GRAP2	

**Table 5:** The predicted relationship between gastric cancer and anandamide.

Link between gastric cancer and B-list	B-list	Link between anandamide and B-list
AKT signaling		Anandamide-inhibitory action
miR-101	cox-2	Inflammatory pain
Ursolic Acid	(PTGS2)	Prostaglandin E2 ethanolamide
Wnt/beta-Catenin Signaling		Spinal nociception
Protease-activated receptor-2		Endocannabinoid metabolism
Anoikis and apoptosis	p38	Mitochondrial biogenesis
MMP-9 suppression	(MAPK14)	MAPK phosphorylation
ER stress		Ca^2+^ elevation
RUNX3-mediated inhibition		CB1-dependent Akt signaling
miR-10b	AKT	Platelets extension
Regulation of SHP2	(AKT)	Suppression of nociception
Apoptosis and metastasis		Apoptosis and migration
Helicobacter pylori Virulence	JNK	Axon regeneration inhibition
DDP-resistance	(MAPK8)	IL-10 production
Invasion and metastasis		CB2 receptors
Cell cycle progression	Chk1	Cdk2 inhibition
Transforming growth factor-beta1	(CHEK1)	Cell cycle arrest

### Detection of the effect of anandamide on gastric cancer cells

**Figure 2:**
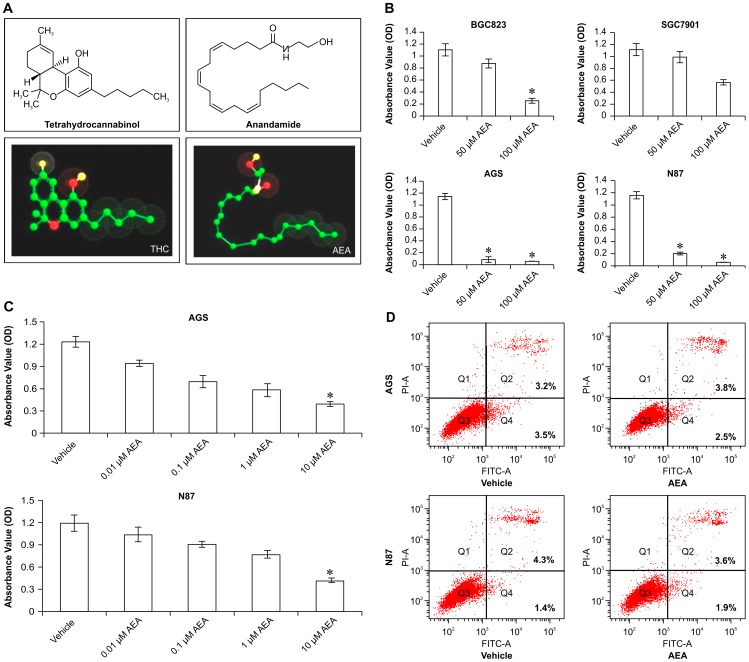
The effect of anandamide on gastric cancer cells.A.The structures of tetrahydrocannabinol and anandamide. Anandamide functional groups include amides, esters, and ethers of long-chain polyunsaturated fatty acids, and structurally share critical pharmacophores with D-9-tetrahydrocannabinol (THC). B.Anandamide treatment inhibits the proliferation of gastric cancer cells. BGC823, SGC7901, AGS, and N87 cells were treated with synthetic anandamide at concentrations of 0, 50, and 100 µM for 24 h. The proliferation rate was detected by the MTT assay. Cells treated with vehicle of ethanol were employed as a control. Data are presented as mean ± SD (n = 3), * indicates *p<0.01* compared with control. C.Anandamide treatment inhibited the proliferation of AGS and N87 cells. AGS and N87 cells were treated with synthetic anandamide with increasing concentrations of 0, 0.01, 0.1, 1, and 10 µM for 24 h. The proliferation rate was detected by MTT assay. Data are presented as mean ± SD (n = 3). * indicates *p<0.01* compared with control. D.The effect of anandamide on apoptosis in AGS and N87 cells. AGS and N87 cells were treated with anandamide at a concentration of 10 µM for 24 h. Apoptosis were analyzed by flow cytometry. Cells treated with vehicle were used as a control.

Anandamide, as a member of the endocannabinoid family, also known as *N*-arachidonoylethanolamine or AEA, was shown to activate 2 distinct G protein-coupled cannabinoid receptors, the cannabinoid receptor type 1(CB1) and the cannabinoid receptor type 2(CB2)[12]. Anandamide is synthesized from N-arachidonoyl phosphatidylethanolamine (NAPE) and its degradation is primarily catalyzed by the fatty acid amide hydrolase (FAAH) enzyme, which also catalyzes the downstream conversion of anandamide into ethanolamine and arachidonic acid. The structure of anandamide includes the functional groups of amides, esters, and ethers of long-chain polyunsaturated fatty acids [13]. These compounds exhibit “cannabimimetic activity”; in other words, they function as “D-9-tetrahydrocannabinol(THC) mimetics”, the active ingredient of Cannabis. The anandamide structure shares critical pharmacophores with THC[14] (Figure 2A).

To explore the effect of anandamide on the proliferation of gastric cancer cells, BGC823, SGC7901, AGS, and N87 were treated with increasing concentrations of synthetic anandamide. Subsequently, the proliferation of these cell lines was detected by MTT. Results demonstrated that anandamide strongly reduced the proliferation rates of these 4 gastric cancer cell lines in a dose-dependent manner, with statistically significant reductions (*p <0.01*) at 100 µM. Moreover, the AGS and N87 cell lines displayed a more sensitive and dramatic decrease (*p <0.01*) even at 50 µM concentration (Figure 2B). Furthermore, we examined the effect of anandamide at lower concentrations on cell proliferation in gastric cancer cells. As shown in Figure 2C, a significant, dose-dependent decrease (*p <0.01*) in proliferation of AGS and N87 cells was observed with an inhibition. However, anandamide at lower concentrations produced little effect on BGC823 and SGC7901 cell lines (data not shown). Therefore, AGS and N87 cell lines were more sensitive to anandamide than BGC823 and SGC7901 cell lines.

Anandamide reduced the growth ability of AGS and N87 cells, while it had little effect on apoptosis in AGS and N87 cells (Figure 2D). Therefore, there must be other reasons that led to the decreased proliferation.

### Effect of anandamide treatment on cell cycle distribution of gastric cancer cells

**Figure 3:**
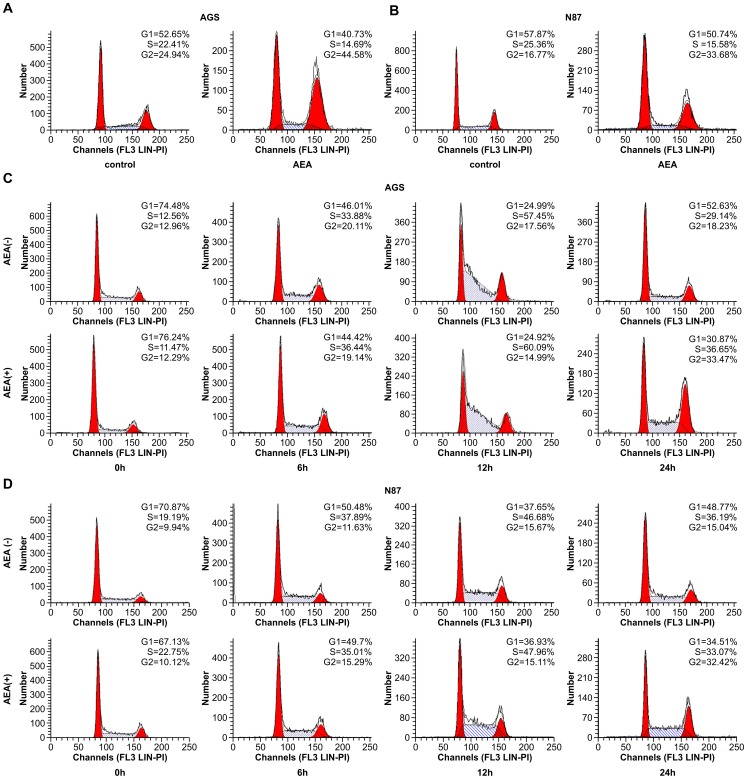
Effect of anandamide on cell cycle distribution of gastric cancer cells. A. The AGS cells at the period logarithmic growth were incubated with 10 µM anandamide or ethanol solvent for 24 h. The cell cycle phase was analyzed with flow cytometry. Cells treated with anandamide experienced a significant G2 block. B. N87 cells were treated with anandamide (10 µM) for 24 hand then halted in the G2 phase of cell cycle. The control cells were treated with ethanol solvent. The cell cycle phase was analyzed with flow cytometry. C. The AGS Cells arrested at G1 phase by serum starvation were released at t  =  0h and incubated with anandamide(10 µM) or ethanol. The cells were collected at 6h, 12h, and 24 h, and DNA contents were analyzed by flow cytometry. The percentage of cells in each phase of cell cycle displayed anandamide causing G2/M phases at 24h. Graphs are representative of 2 independent experiments with similar results. D. The N87 cells were synchronized in G1 phase by serum starvation and were released at t = 0, simultaneously they were treated with anandamide(10 µM) or ethanol. The cells were harvested at 6h, 12h, 24h and prepared for flow cytometry analysis. Graphs are representative of 2 independent experiments with similar results.

To further examine the characteristics of growth inhibition by anandamide, the cell cycle phase was detected by flow cytometry. The AGS and N87 cells at the period logarithmic growth were incubated with 10 µM anandamide or ethanol solvent for 24 h, then halted in the G2 phase of cell cycle (Figure 3A and 3B). To investigate the effect of anandamide on cell cycle progression in greater detail, AGS and N87 cells were synchronized in the G1 phase by serum starvation. The cell cycle was analyzed when cells were released from this blockade with complete medium in the presence or absence of anandamide. Cells treated with ethanol solvent in place of anandamide were employed as controls. As seen in Figure 3C, AGS cells were synchronized with more than 70% G1 phase DNA content after they were grown in free-serum culture. By 6 h, approximately 46% of control cells were in G1 phase, and by 12 h, the majority (57.45%) were in S phase. Between 12 and 24 h, the cells were in G2/M phase and underwent rapid mitosis, and by 24 h, they entered the next cell cycle. However, when the cells were treated with anandamide after release from the G1 phase, 33.47% of the treated cells were arrested in G2/M phase for up to 24h, and showed a delayed entry into the next cell cycle. A similar trend was also observed in N87 cells (Figure 3D). Taken together, these results indicate that anandamide treatment caused the G2/M arrest observed in AGS and N87 cells.

### Effect of anandamide on cell cycle regulators

**Figure 4:**
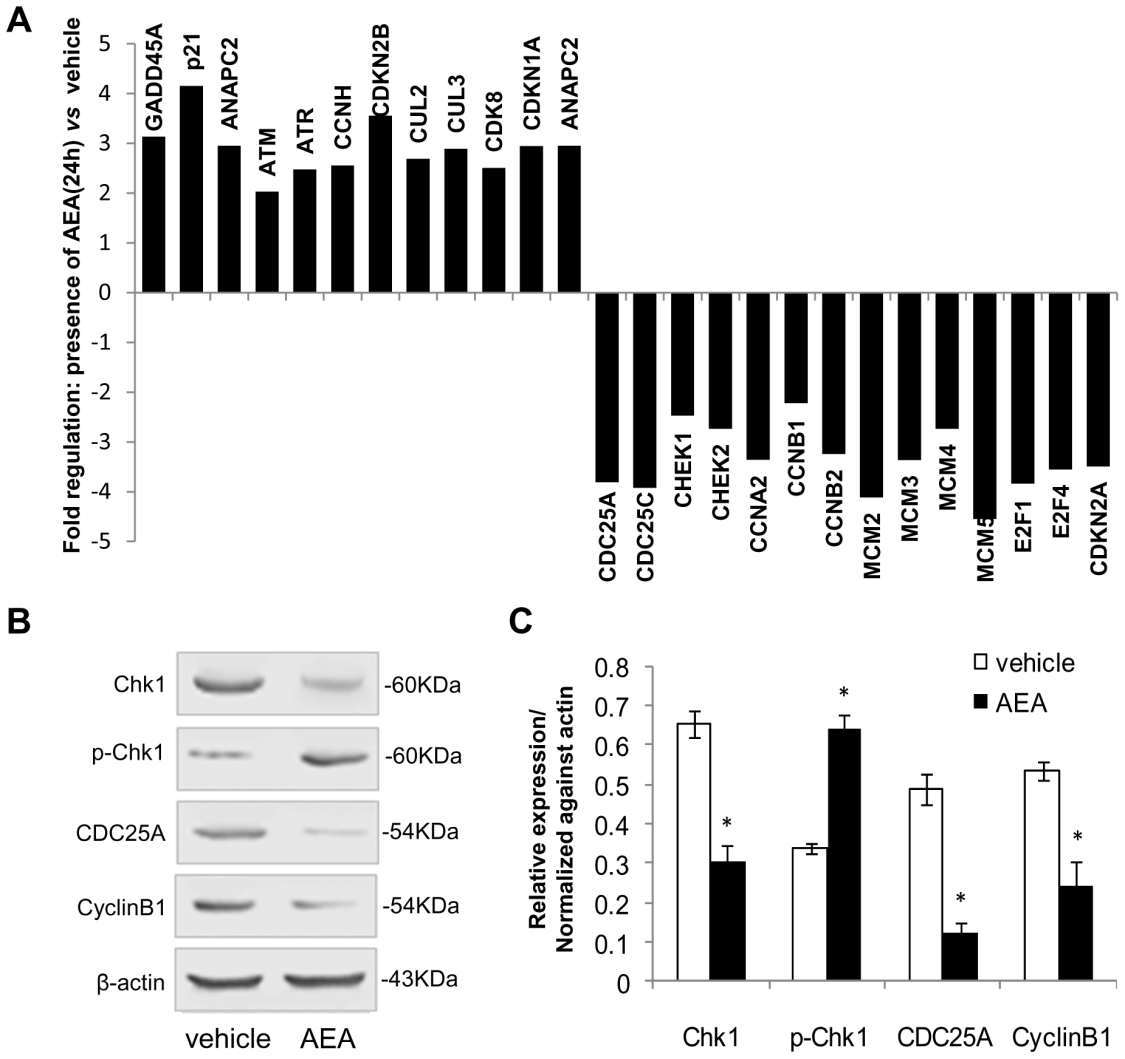
The effect of anandamide on cell cycle regulators. A. Analysis of differential gene expression profiling in the AGS cells with the presence and absence of anandamide. Data were obtained from three samples each group. The differential gene with average Changes(fold >2) after exposure to anandamide (10 µM) for 24 h were picked up, the graphs show the fold changes of these cell cycle related genes. B. Effect of anandamide on the expression of the cell cycle regulators. Cells were harvested after anandamide (10 µM) or ethanol solvent treatment for 24h and analyzed by western blotting with antibodies against Chk1, p-Chk1, CDC25A, and CyclinB1. C. The results of western blotting were normalized against β-actin. Fold changes of these proteins were determined by comparison with levels measured in control ethanol treated cells. Data are presented as mean ± SD (n  =  3), * indicates *p<0.05* compared with control.

In order to analyze the molecular mechanisms responsible for increased cell cycle arrest caused by anandamide, we used 2 different RT2 Profiler PCR arrays(SA Biosciences, QIAGEN) to determine the expression levels of genes involved in the regulation of cell cycle. Genes with expression fold changes between cells treated with anandamide and vehicle larger than 2 were selected as differential genes. The results showed that AGS cells treated with anandamide for 24 h displayed an overall increase in the expression of G2/M arrest related genes including GADD45 and p21^waf^ and an apparent decrease in G2 cell cycle regulators including CHEK1,CHEK2, CDC25, CCNA2, CCNB1, CDKN1A, CDKN2A, etc.(Figure 4A). To further investigate whether anandamide caused cell cycle arrest by G2/M checkpoint control, we performed a western blot analysis of AGS cells following anandamide treatment for 24h. Cells treated with ethanol solvent in place of anandamide were employed as controls. As shown in Figure 4B and 4C, anandamide caused an increase of Chk1 phosphorylation and decreased the expression levels of Cyclin B1 and CDC25A compared to the observations in control cells. These data suggested that anandamide treatment led to increased activity at G2/M checkpoints.

## Discussion

Using Swanson's literature-based discovery methodology, investigators may determine novel relationships between seemingly unrelated fields, and resurrect previously published but neglected hypotheses [15]. In the present study, we simulated Swanson's literature-based discovery methodology in 2 fields of medical research literature that have not been bibliographically connected: gastric cancer and psychology. After a 2-step discovery process, anandamide was selected and predicted to be linked to gastric cancer. Next, the potential relationship between gastric cancer and anandamide was confirmed by follow-up experimentation. Hence, we successfully simulated literature-based discoveries because the hypotheses generated a novel relationship that has been proven to be reliable.

Gastric cancer is a multifactorial disease, with numerous causative variables having been reported, including *H. pylori*, high-sodium diets, drinking, and smoking [16]. Previous studies in our laboratory have specialized in gastric cancer; therefore the objective of this study was to find new knowledge in the gastric cancer research field. This approach, using Swanson's literature-based discovery, led to the identification of anandamide, a member of the endocannabinoids. Anandamide has been reported to exhibit anti-proliferation properties when treating certain types of cancers, including neuroblastoma, prostate carcinoma and melanoma [17]–[20]. Mayato [21] also reported research on anandamide in gastric cancer. They investigated the inhibitory effect of anandamide on gastric cancer cells and clarified the function of anandamide to enhance the cytotoxic effect of paclitaxel, and their conclusions regarding the effects of anandamide on cell growth are consistent with results of this study. However, some genes (CHEK1, CDKN1A, CDKN2A, CCNB1, etc.) from the B-terms of the close discovery were cell cycle regulators, suggesting that anandamide might be related to cell cycle in gastric cancer. As expected, real-time PCR and western blot assays detected that the expressions of those cell cycle regulators were significantly altered following anandamide treatment. Flow cytometry assays further confirmed that anandamide induced G2/M cell cycle arrest in gastric cancer cells through active G2/M checkpoints. This represents the first time that the cell cycle redistribution was detected in gastric cancer cells after being treated with anandamide directly and separately. Additionally, the results indicated that the B-terms could potentially function to mediate the effectors between the disease and the discovery targets.

For biological investigators, keeping up-to-date with current published research is a critical component of any investigator's job description, and nearly every published article is an opportunity to find novel links between drug and disease. However, the current volume of available biological science is enormous. Using the informed traditional search, investigators may observe limited links between a drug/molecule and a disease, but may not recognize the larger environment through which the relationships operate, nor identify other potential relationships within that environment. Therefore, information sciences and retrieval are very useful tools for biological scientists. Swanson's literature-based discovery is focused on resurrecting previously published but neglected hypotheses. If a direct connection that seems to be neglected is detected, then the work of resurrection turns out to be of analyzing pathways or mechanisms that might not be known. In other words, Swanson's literature-based discovery methodology not only mines data for possible interactions between disease and disease, disease and drug, or disease and molecule, but also provides us with the potential to observe the larger background behind these direct links, like the molecular pathway network in our study suggesting a possible link between gastric cancer and anandamide action. By analyzing data in this context, we obtained the interactions and the mechanisms between seemingly unrelated topics and their clinical importance and significance. Therefore, swanson' s literature-based discovery is an effective tool to seek connected existing knowledge from empirical results by bringing to light relationships that are implicated and yet "neglected" (Marc Weeber, 2001).

We also encountered some challenges in the discovery process. One problem was with the MeSH terms, themselves, and the additional visual inspection was required following extraction using our custom Java applet. In the open discovery process, we often are unable to recognize abbreviations that can represent different words, and we therefore ignored them despite their potential importance. Additionally, some of the terms collected were auxiliary words, resulting in redundancy. Moreover, there were instances when synonyms were initially counted separately because of their spelling, and these duplications had to be resolved manually. Despite these issues, the literature-based discovery methodology increase clearly presents an advantage in the process of discovering new relationships from diverse fields of research.

Swanson originally developed the literature-based discovery methodology and has been refining this process for 30 years with the aim of helping investigators discover unknown or unidentified relationships between studies; a problem that has remained poorly understood until now. Using this Discovery methodology, we found a potential interaction between anandamide and gastric cancer, which was deeply buried in the vast amounts of available data, and which has now been reliably confirmed by experimentation. In our study, anandamide inhibited the proliferation of gastric cancer cells and mediated G2/M cell cycle arrest by altering the expression of the cell cycle regulators. This relationship has been neglected for many years before we identified the unknown relationship. Our study demonstrates that Swanson's literature-based discovery methodology is a useful way to assist investigators in uncovering novel interactions between studies by efficiently scanning large amounts of literature to strengthen their initial hypotheses.

Due to the rapidly increasing numbers of medical publications and electronic databases that are available, it is now necessary to find novel ways to access the relationships between different studies [22]. In this situation, Swanson developed a literature-based discovery methodology to extract information and predict possible relevance among articles which are not bibliographically connected. This methodology has significance, not only for individual investigators but also for the field of science as a whole. Medical specialists can retrieve articles and find ignored relationships more efficiently, and doing so may lead to greater discoveries relating to public health, all with the help of this discovery procedure. Swanson's literature-based discovery methodology is an invaluable discovery tool for scientists.
